# Multi-omics elucidation of yellow aril coloration in litchi (*Litchi chinensis* Sonn.) cultivar ‘Jianjianghongnuo’: coordinated downregulation of flavonoid and carotenoid biosynthetic pathways drives pigment dynamics

**DOI:** 10.3389/fpls.2025.1669458

**Published:** 2025-10-06

**Authors:** Mianqiao Pan, Han Wang, Zhenchen Ning, Xiaoqian Yu, Hecheng Liu, Xiaomei Huang, Houbin Chen, Zuanxian Su

**Affiliations:** ^1^ Guangdong Litchi Engineering Research Center, College of Horticulture, South China Agricultural University, Guangzhou, China; ^2^ Guangdong Laboratory of Lingnan Modern Agriculture, Guangzhou, China

**Keywords:** litchi, yellow aril, coloration mechanism, transcriptomics, metabolomics, flavonoid, carotenoid

## Abstract

Litchi (*Litchi chinensis* Sonn.) arils are predominantly white or cream-colored, with yellow-pigmented cultivars being rare and poorly characterized. The ‘JJHN’ cultivar exhibits a distinctive yellow aril that progressively attenuation during fruit development, providing an ideal model to investigate the biochemical and molecular basis of yellow pigmentation in litchi. In this study, we integrated phenotypic evaluation, widely targeted and carotenoid-targeted metabolomics, and transcriptomic analyses at ‘JJHN’ arils across fruit development. The color shift from orange-yellow to pale yellow during ripening, as indicated by declining CIE b* values, was accompanied by significant reductions in total flavonoid and carotenoid contents. Widely targeted metabolomics profiling revealed that aurones (e.g., aureusidin 4,6-diglucoside) and chalcones (e.g., naringin chalcone) were the primary yellow pigments. Targeted carotenoid analysis identified violaxanthin as the predominant carotenoid during early development, accounting for 62.4% of total carotenoids at stage Y2, but decreasing sharply and becoming undetectable at maturity. Transcriptomic data showed coordinated downregulation of structural genes involved in flavonoid (e.g., *CHS*, *CHR*, *CHI*) and carotenoid (e.g., *ZEP*, *AAO3*) biosynthesis, aligning with the observed pigment depletion. Weighted gene co-expression network analysis (WGCNA) identified a “turquoise” module positively correlated with flavonoid content, comprising transcription factors from MYB, bHLH, bZIP, NAC, and DOF families that possessed predicted binding sites in pigment-related gene promoters. Collectively, these findings demonstrate that the yellow aril phenotype of cv. ‘JJHN’ is regulated by a dual pigment system—aurones/chalcones and violaxanthin—whose developmental depletion is transcriptionally controlled by a complex TF network. This study provides novel insights into the regulation of fruit pigmentation and lays a foundation for breeding litchi cultivars with yellow arils.

## 1 Introduction

Litchi (*Litchi chinensis* Sonn.) is a subtropical fruit tree cultivated in over 20 countries, with the largest planting area located in South China (Hu et al., 2022). Owing to its distinctive flavor and high nutritional value, litchi has become one of the most attractive tropical and subtropical fruits on the international market. In most cultivars, the aril appears waxy white or milky white. However, yellow-ariled cultivars, such as ‘Jianjianghongnuo’ (cv. ‘JJHN’), remain scarce, and the biochemical mechanisms underlying yellow pigmentation in litchi arils are still poorly understood.

Plant color formation involves diverse biochemical compounds, with flavonoids, carotenoids, and betalains constituting the three primary pigment classes (Zhao et al., 2022).

Flavonoid compounds are commonly found in flowers, fruits, leaves, and seeds, where they determine color, aroma, and flavor characteristics (Vicente and Boscaiu, 2018). More than 10,000 flavonoids have been identified and classified into at least 10 chemical groups (Liu et al., 2021). Among these, anthocyanins are responsible for red, magenta, orange, pale yellow, blue, and violet colors, while aurones and chalcones typically produce yellow coloration (Grotewold, 2006). And flavones and flavonols are colorless or very pale yellow (Liu et al., 2021). Flavonoid biosynthesis begins with the deamination of phenylalanine by phenylalanine ammonia-lyase (PAL), followed by cinnamate 4-hydroxylase (C4H), leading to the formation of chalcone—the central precursor of the flavonoid pathway. The first committed step is catalyzed by chalcone synthase (CHS), which condenses one molecule of p-coumaroyl-CoA with three molecules of malonyl-CoA to produce naringenin chalcone. This intermediate is then converted to the flavanone naringenin by chalcone isomerase (CHI). Sequential actions of downstream enzymes result in the biosynthesis of diverse flavonoid classes, including flavanones, dihydroflavonols, and anthocyanins (Hassani et al., 2020; Liu et al., 2021; Chen et al., 2023b).Flavonoid biosynthesis is tightly regulated by biosynthetic enzymes and regulatory transcription factors (TFs) (Fenn and Giovannoni, 2021), with several TF families reported to be involved, including WRKY, Dof, MADS-box, bZIP, MYB, bHLH, WD40, and NAC (Qiu et al., 2019; Corso et al., 2020; Qian et al., 2021).

Carotenoids, a group within the terpenoid family, are synthesized in plastids, where they contribute to the vivid yellow, orange, and red hues observed in plant tissues. In addition to their role in pigmentation, carotenoids are vital for photosynthesis, particularly in light capture and protection against photo-oxidative damage. They also serve as metabolic precursors for key phytohormones, including abscisic acid and strigolactones (Kou et al., 2021; Li et al., 2022a). Their biosynthesis begins with the universal five-carbon building blocks—isopentenyl diphosphate (IPP) and its isomer dimethylallyl diphosphate (DMAPP)—originating from the methylerythritol 4-phosphate (MEP) pathway. These precursors combine to form geranylgeranyl diphosphate (GGPP), from which the first colorless carotenoid, phytoene, is produced via the condensation of two GGPP molecules. The early biosynthetic steps are catalyzed by enzymes encoded by *GGPS*, *PSY*, *PDS*, *Z-ISO*, *ZDS*, and *CRTISO*. Phytoene is subsequently transformed into a variety of carotenoid compounds through reactions such as desaturation, cyclization, hydroxylation, and epoxidation, involving key genes like *LCYE*, *LCYB*, *CHYB*, *ZEP*, *VED*, *NXS*, *CYP97A*, and *CYP97C* (Wolters et al., 2010; Li et al., 2024; Cheng et al., 2025). The core biosynthetic network underlying carotenoid metabolism has been thoroughly characterized across multiple plant taxa (Sun et al., 2018). Lutein serves as the main pigment responsible for yellow coloration in rapeseed flowers (Liu et al., 2020), while the orange color of sweet potatoes results from lutein, neoxanthin, capsanthin, and β-carotene (Ducreux et al., 2005). Such coloration differences are directly controlled by variations in biosynthetic gene expression, leading to diverse carotenoid compositions across species. Additionally, the plant hormone abscisic acid (ABA) represents one of the major products of carotenoid metabolism and has been extensively studied for its role in regulating plant growth and stress responses (Sun et al., 2022). The direct precursors of ABA include xanthophylls, violaxanthin, and neoxanthin, with the first committed step in ABA biosynthesis being the oxidative cleavage catalyzed by 9-cis-epoxycarotenoid dioxygenases (NCED) (North et al., 2007).

Betalains, exclusive to Caryophyllales plants, contribute to red and yellow hues. Red beet derives its color from betanin and 2-decarboxybetanin, while yellow prickly pear fruit is colored by indicaxanthin (Polturak and Aharoni, 2018).

Previous studies have primarily focused on anthocyanin-driven color formation in litchi pericarps; however, comprehensive analyses have not yet been conducted to elucidate the mechanisms underlying yellow aril formation in litchi. Therefore, this study focused on yellow aril formation in cv. ‘JJHN’ by integrating phenotypic, widely targeted metabolomics, carotenoid-targeted metabolomics, and transcriptomics analyses across different fruit developmental stages. This multi-dimensional approach aimed to elucidate the regulatory networks and metabolic pathways underlying yellow aril pigmentation, thereby providing a theoretical foundation for the genetic improvement and breeding of yellow-ariled litchi cultivars.

## 2 Materials and methods

### 2.1 Plant materials

Fruits of *Litchi chinensis* cv. ‘Jianjianghongnuo’ (cv. ‘JJHN’) were collected from trees cultivated in the Ziyi Gongyuan Orchard, Gaozhou County, Guangdong Province, China. Sampling was performed at 7-day intervals, beginning 49 days after anthesis and continuing until full maturity, corresponding to stages Y1 through Y5. Arils were immediately separated, flash-frozen in liquid nitrogen, and stored at -80 °C for subsequent analyses.

### 2.2 Color, total flavonoid and total carotenoid content measurement

The colorimetric properties of arils were quantified across five developmental stages using a CIELab-calibrated digital colorimeter (model NR110, 3nh Technology Co., Ltd., Shenzhen, China). Measurements included three chromatic coordinates: L* (lightness: 0 [absolute black] to 100 [diffuse white]), a* (green-red axis: -128 [greenness] to +127 [redness]), and b* (blue-yellow axis: -128 [blueness] to +127 [yellowness]) (Zhou et al., 2024). Total flavonoid content (TFA) was determined using the BC1335 assay kit (Beijing Solarbio Science & Technology Co., China) following the manufacturer’s protocol. Fresh aril samples were subjected to ultrasonic extraction in a 60 °C water bath at a frequency of 300 Hz for 30 minutes. The extracts were then centrifuged at 12,000 rpm for 10 minutes at 25 °C, and the absorbance of the supernatant was measured at 470 nm (Gong et al., 2023). Total carotenoid content (TLC) was quantified using the BC4335 assay kit (Beijing Solarbio Science & Technology Co., China) according to the manufacturer’s instructions. Fresh aril samples were extracted with 80% acetone for 3 hours, and the absorbance of the resulting solution was measured at 440 nm (Wu et al., 2024).

### 2.3 Widely targeted metabolomics

For flavonoid analysis, we employed widely targeted metabolomics, a powerful approach that enables the simultaneous relative quantification of diverse secondary metabolites with distinct structures. Aril samples were ground for 1.5 minutes at 30 Hz using a tissue mill (MM400, Retsch, Berlin, Germany). Subsequently, 100 mg of the resulting powder was extracted overnight at 4 °C with 1.0 mL of 70% methanol. After centrifugation at 10,000 × g for 10 minutes, the supernatant was collected and filtered through a 0.22 μm microporous membrane. The resulting extracts were transferred into vials and stored for subsequent analysis using ultra-high-performance liquid chromatography coupled with tandem mass spectrometry (UHPLC–MS/MS), based on a widely targeted metabolomics approach.

Metabolite profiling of litchi aril samples was performed using a liquid chromatography–electrospray ionization–tandem mass spectrometry (LC–ESI–MS/MS) system by MetWare (Wuhan, China). Detailed procedures are provided in **Supplementary Methods S1** (Deng et al., 2023; Jiang et al., 2023). Differentially accumulated metabolites (DAMs) were identified based on the criteria of variable importance in projection (VIP) > 1, fold change ≥ 2 for upregulated metabolites, and fold change ≤ 0.5 for downregulated metabolites. Both widely targeted metabolomic and carotenoid-targeted metabolomic analysis was conducted by Wuhan MetWare Biotechnology Co., Ltd. (Wuhan, China; https://www.metware.cn) (Zheng et al., 2022).

### 2.4 Carotenoid-targeted metabolomic analysis

For carotenoid analysis, we used targeted metabolomics with absolute quantification, as this class of compounds requires specific extraction procedures and standard curves for accurate concentration measurement due to their instability and the critical importance of their absolute levels for color presentation. Plant materials were freeze-dried and finely ground using a ball mill. After the addition of an appropriate internal standard, 50 mg of the sample powder was extracted with a solvent mixture of n-hexane, acetone, and ethanol (1:1:2, v/v/v) containing 0.01% butylated hydroxytoluene (BHT, g/mL) as an antioxidant. The extraction was performed twice. Supernatants from both extractions were combined, evaporated to dryness, and reconstituted in 100 μL of a methanol/methyl tert-butyl ether (MTBE) mixture (3:1, v/v). The final solution was filtered through a 0.22 μm membrane and transferred to amber vials for UPLC–MS/MS analysis. Detailed procedures are provided in **Supplementary Methods S2**.

### 2.5 Transcriptome sequencing and analysis

Total RNA was extracted from litchi arils using the CTAB-PBIOZOL method followed by ethanol precipitation, with three biological replicates per stage. mRNA libraries were constructed for each sample and sequenced using the Illumina NovaSeq 6000 platform. Transcriptome data processing and analysis followed previously established protocols (Li et al., 2023). Clean reads were aligned to the *Litchi chinensis* cv. ‘Feizixiao’ reference genome (http://www.sapindaceae.com, accessed on 10 April 2024) using HISAT2 for index building and alignment (Guo et al., 2022; Hu et al., 2022).

Differential gene expression analysis was conducted using DESeq2 (Love et al., 2014), with thresholds set at p-value < 0.05 and |log_2_(fold change)| > 1 to identify differentially expressed genes (DEGs). All analyses were performed in R version 3.5.1. Gene Ontology (GO) enrichment was carried out using the clusterProfiler package (Ma et al., 2022), and visualized with ggplot2. KEGG pathway enrichment results were used to construct regulatory network diagrams. Inter-omics Pearson correlation networks were computed in R and visualized via Metware Cloud (Li et al., 2022b).

### 2.6 WGCNA analysis

Weighted gene co-expression network analysis (WGCNA) was conducted using the WGCNA package in R (Langfelder and Horvath, 2008). Genes with low or undetectable expression levels (defined as a total TPM value < 1 across all samples) were excluded, resulting in 17,215 genes retained for downstream analysis. Network construction parameters were set as follows: soft-thresholding power = 8, minimum module size = 30, and merge cut height = 0.25. Module eigengenes (MEs) were correlated with flavonoid content to identify biologically relevant modules. An unsigned co-expression network was constructed, and transcription factor (TF)–target gene relationships were preliminarily inferred. Putative DNA-binding motifs were identified using the PlantTFMotifShift plugin in TBtools (Chen et al., 2023a), with *Arabidopsis thaliana* as reference. Motif scanning was subsequently performed using FIMO (Find Individual Motif Occurrences) from the MEME Suite (Nystrom and McKay, 2021). Genes containing statistically significant motif matches (P-value < 1.0 × 10^-4^) within their promoter regions (upstream 2 kb) were considered potential targets of the corresponding TFs. The network was analyzed using Cytoscape and visualized using the Metware Cloud (https://cloud.metware.cn).

### 2.7 qRT-PCR analysis

Total RNA isolated as described in Section 2.5 was reverse-transcribed into cDNA using the HiScript II Reverse Transcriptase kit (Vazyme Biotech, Nanjing, China). Quantitative real-time PCR (qRT-PCR) was conducted on a LightCycler 480 II system (Roche, Germany) with ChamQ Universal SYBR qPCR Master Mix (Vazyme Biotech, Nanjing, China), following the manufacturer’s protocol. *LcActin* (GenBank accession no. HQ615689) served as the internal reference gene. Relative transcript levels were quantified using the 2^−ΔΔCT^ method (Li et al., 2023). All primers used were designed with TBtools II (Chen et al., 2023a).

### 2.8 Statistical analysis

The statistical analysis was performed with SPSS using one-way analysis of variance (ANOVA). The results were expressed as means ± standard error (SE), and differences among samples at p < 0.01 were regarded as extremely significant. The heatmap and Venn diagram were prepared using the Metware Cloud (https://cloud.metware.cn).

## 3 Results

### 3.1 Progressive attenuation of yellow coloration in ‘JJHN’ arils correlated with diminished flavonoid and carotenoid contents during fruit development

**Figure 1:**
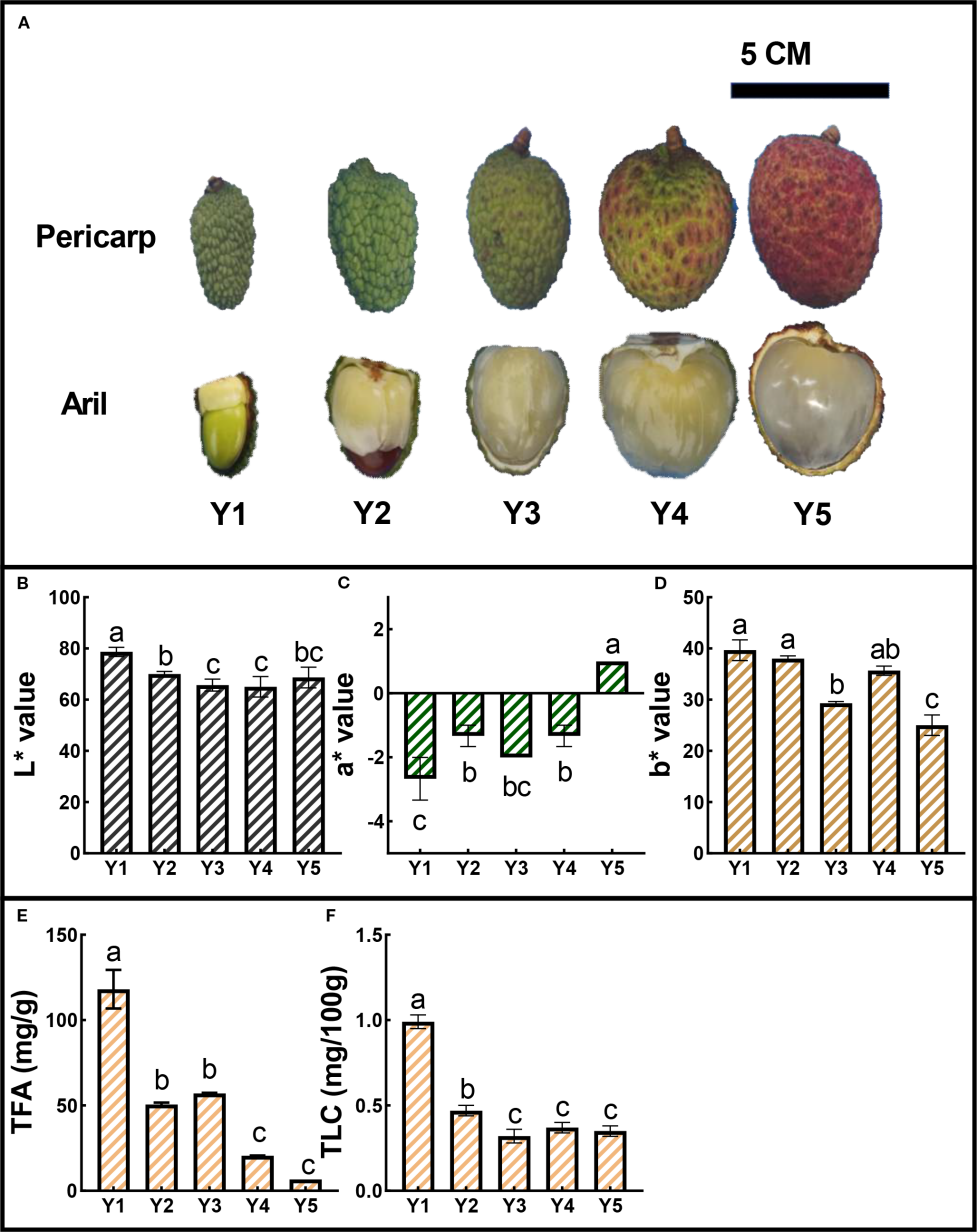
Color dynamics and pigment content of ‘JJHN’ across five developmental stages (Y1–Y5). **(A)** Morphological changes in arils and pericarps. Scale bars = 5 cm. **(B-D)** CIELab coordinates (L*, a*, b*) of ‘JJHN’ arils. **(E, F)** Total flavonoid content (TFA) and total carotenoid content (TLC) in ‘JJHN’ arils, expressed as mg/g fresh weight (FW) and mg/100 g FW, respectively. Data are presented as mean ± standard error (SE). Significant differences among developmental stages were determined by one-way ANOVA followed by Duncan’s multiple range test, and are indicated by different letters (p < 0.05).

To investigate the mechanisms underlying the yellow aril coloration in litchi, we selected the representative cultivar ‘JJHN’, which consistently exhibits yellow arils throughout fruit development. During the early developmental stages, the arils displayed an orange-yellow hue. From stage Y2 to Y4, this coloration progressively faded, resulting in a pale-yellow appearance at maturity (Y5), concurrent with the onset of red pigmentation in the pericarp (**Figure 1A**). To quantitatively characterize aril color changes from stages Y1 to Y5, we measured L*, a*, and b* values. The L* values, representing brightness, ranged from 65 to 78, indicating moderate luminosity across developmental stages (**Figure 1B**). The a* values (red-green axis) showed minimal fluctuation, remaining between -3 and 2 (**Figure 1C**). In contrast, the b* values remained stable from Y1 to Y2, but declined notably at Y3, suggesting a transient reduction in yellow chroma at this intermediate stage. After a partial recovery at Y4, b* values dropped sharply again at Y5. This pattern suggests a reduction in yellow intensity during development (**Figure 1D**). To examine the potential biochemical basis for these color changes, we quantified TFA and TLC. Both exhibited significant decreasing trends throughout fruit maturation. TFA declined markedly from early to late stages (**Figure 1E**), paralleled by a steady reduction in total carotenoid levels (**Figure 1F**). These results suggest that the attenuation of yellow pigmentation in ‘JJHN’ arils is closely associated with the degradation or reduced accumulation of TFA and TLC during fruit development.

### 3.2 Widely targeted metabolomics revealed a coordinated decline in flavonoid metabolites as the pigment basis of yellow color dynamics

To elucidate the metabolic basis underlying aril pigmentation in ‘JJHN’ litchi, we systematically profiled flavonoid composition across developmental stages, identifying distinct patterns of differential accumulation among key pigment-related compounds. Among the 2,012 differentially accumulated metabolites (DAMs) (Dataset S1) identified during aril development, 429 were classified as differentially accumulated flavonoids (DAFs) (Dataset S2), comprising 139 flavonols, 105 flavones, 45 flavanones (or dihydroflavones), 38 flavanols, 26 chalcones, 19 isoflavones, 17 anthocyanins, 9 flavanonols (or dihydroflavonols), and 4 aurones. The total DAFs contents showed an overall downward trend from Y2 to Y5, including chalcones, aurones, flavanonols, anthocyanins, flavones, flavanols, and isoflavones. Particularly, flavonols and flavanones exhibited a slight accumulation increase between Y3 and Y4 (**Table 1**).

**Table 1:** Relative quantification of DAFs contents in ‘JJHN’ arils during fruit development.

Content (×10^6^)	Y2	Y3	Y4	Y5
Flavonols	613.959	411.588	453.391	247.236
Flavones	297.050	162.879	99.402	56.982
Flavanols	169.626	80.600	61.378	26.503
Anthocyanidins	144.635	110.598	108.315	67.435
Chalcones	129.982	58.438	55.208	29.443
Flavanonols	128.601	65.559	49.678	28.119
Flavanones	121.690	80.517	88.633	43.285
Isoflavones	64.290	30.781	11.509	6.344
Aurones	5.933	4.054	3.741	1.738
Other Flavonoids	73.225	54.540	36.963	17.472
Total DAFs	1748.991	1059.554	968.218	524.555

Yellow pigment-associated compounds exhibited distinct developmental patterns. Among the four aurones, three compounds (cernuoside, maesopsin 6-beta-D-glucopyranoside, and aureusidin 4,6-diglucoside) declined continuously during fruit development, whereas aureusidin 6-glucuronide increased mildly from Y3 to Y4. Within the 26 chalcones, 14 compounds (including xanthohumol, phlorizin chalcone, and trilobatin) decreased consistently, while three compounds (isobavachalcone glucoside, naringin chalcone, and isoliquiritin) increased slightly from Y3 to Y4. Notably, marein exhibited dramatic accumulation (log_2_ FC = 5.0) during this period, and two additional chalcones (okanin-4’-(6’’-O-acetyl) glucoside and 3-Hydroxyphloretin-2’-O-(6’’-O-xylosyl) glucoside) increased progressively from Y2 to Y5. Moreover, both aurones and chalcones demonstrated strong positive correlations with the b* value (R > 0.8) (**Supplementary Table S1**).

Weakly pigmented compounds showed variable patterns. Among the 105 flavones, 74 DAFs (such as O-MethylChrysoeriol-8-C-glucoside and apigenin-7-O-rutinoside) decreased across developmental stages, while 10 increased moderately. Two compounds, 6,8-Di-C-glucopyranosylnaringenin and hispidulin-8-C-(2’’-O-xylosyl) glucoside, increased continuously throughout development. Among the 139 flavonols, 78 DAFs (including gossypetin, myricetin-3-(2’’,3’’-digalloyl) rhamnoside, and kaempferol-3-O-glucuronide) declined, whereas 22 increased to varying degrees. Three flavonols (tribuloside, kaempferol-3-O-rutinoside-7-O-glucoside, and kaempferol-3-O-Gentiobioside-7-O-Rhamnoside) exhibited continuous upregulation.

Anthocyanidins, potential contributors to red pigmentation, showed predominantly declining trends. Ten of the 17 Anthocyanidins DAFs (including cyanidin 3-O-rutinoside and peonidin-3-O-rutinoside) decreased from Y2 to Y5, while three compounds, including delphinidin-3-(p-coumaroyl)-rutinoside-5-glucoside, increased over time.

These findings indicate that aurones and chalcones constitute the primary contributors to yellow pigmentation in ‘JJHN’ litchi arils, with their dynamic accumulation patterns closely correlating with aril color variations. The slight yellow intensification observed from Y3 to Y4 likely results from increased accumulation of five specific compounds: aureusidin 6-glucuronide, isobavachalcone glucoside, naringin chalcone, isoliquiritin, and marein. Flavones and flavonols, as weakly pigmented compounds, likely play secondary roles in fine-tuning yellow coloration during development.

### 3.3 Carotenoid-targeted metabolomics revealed violaxanthin dynamics as the dominant carotenoid contributor to yellow pigmentation

To delineate the metabolic dynamics of carotenoid compounds in ‘JJHN’ arils, carotenoid-targeted metabolomic analysis was performed at three distinct developmental stages (Y2, Y3, and Y5). Biological replicates demonstrated tight clustering patterns (**Supplementary Figure S1**), confirming robust experimental reproducibility. Through LC-MS-based quantitative profiling, 16 carotenoid species were quantified (**Table 2**), with violaxanthin emerging as the predominant carotenoid during early development (5.958 μg/g at Y2), which exhibited progressive stage-dependent depletion, decreasing sharply by 72.2% during the Y2-Y3 transition, and reaching undetectable levels by the Y5 maturation stage. This indicated that carotenoids mainly contribute to the color of ‘JJHN’ arils in the early stage of fruit development, with a minor or even negligible contribution in the middle and late stages.

**Table 2:** Differentially accumulated carotenoids in ‘JJHN’ arils during fruit development.

Carotenoids (μg/g)	Y2	Y3	Y5
violaxanthin	5.958	1.658	0
zeaxanthin	0.769	0.596	0.526
neoxanthin	0.505	0.182	0
lutein	0.483	0.238	0.107
violaxanthin-myristate-caprate	0.431	0.317	0.291
α-carotene	0.328	0	0
fucoxanthin	0.299	0.212	0
β-carotene	0.271	0.123	0
β-cryptoxanthin	0.148	0.095	0
rubixanthin laurate	0.081	0	0
α-cryptoxanthin	0.073	0	0
violaxanthin palmitate	0	0	0.429
antheraxanthin dipalmitate	0	0.098	0
violaxanthin myristate	0	0.194	0
lutein oleate	0	0	0
lutein dimyristate	0	0.081	0

### 3.4 Transcriptomic shifts reveal stage-specific regulation of pigment metabolism in aril development

**Figure 2:**
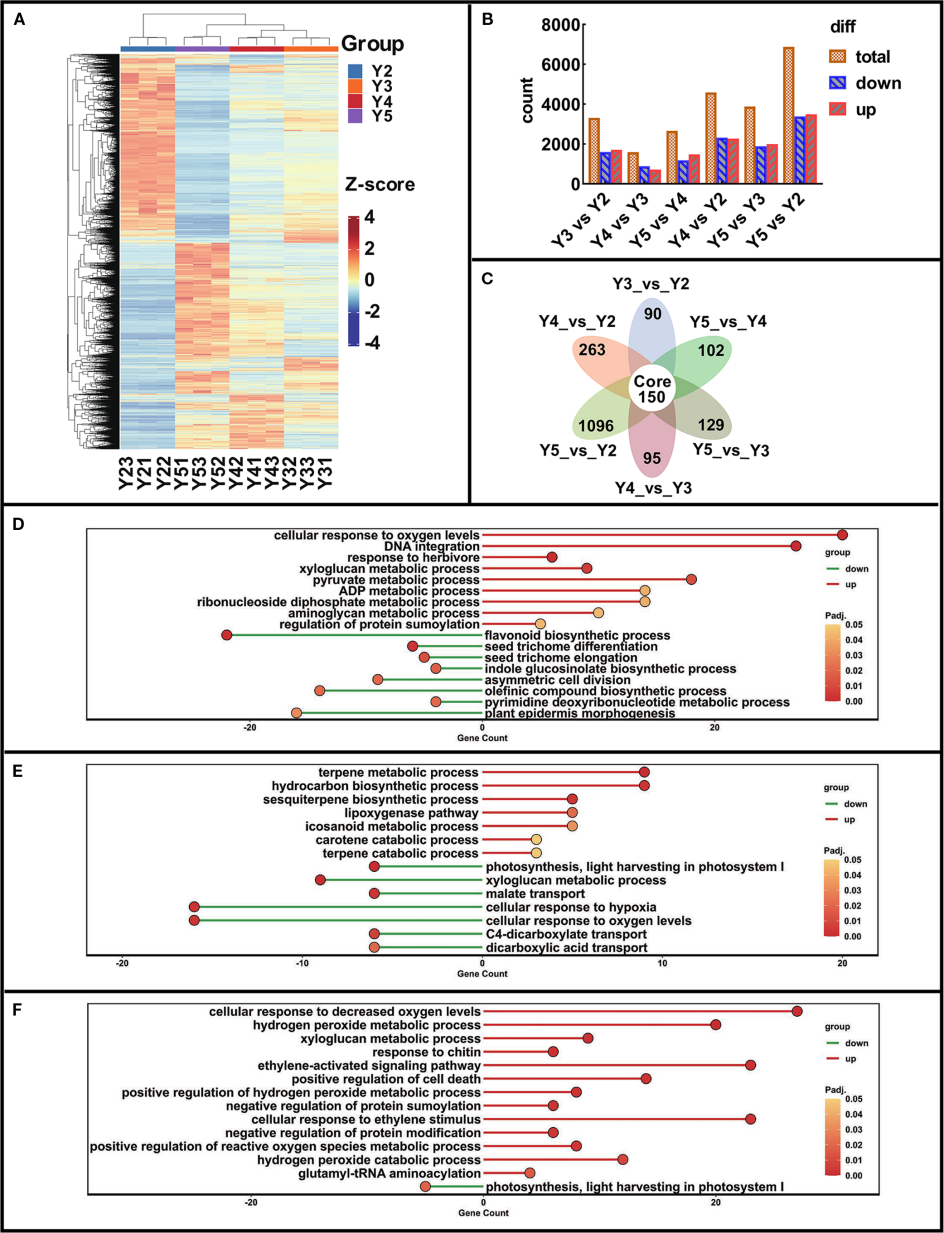
DEGs analysis in ‘JJHN’ arils during fruit development. **(A)** Heatmap of all DEGs. (z-score normalized). **(B)** DEGs count across various comparison groups. **(C)** Venn diagram of DEGs across various comparison groups. **(D-F)** GO Enrichment dotplot of Y3 vs Y2, Y4 vs Y3, and Y5 vs Y4 (bubble size: gene count; color: -log_10_(p-value)).Comparisons of DEGs between developmental stages Y3 and Y2 (Y3 vs Y2), Y4 and Y3 (Y4 vs Y3), Y5 and Y4 (Y5 vs Y4), Y4 and Y2 (Y4 vs Y2), Y5 and Y3 (Y5 vs Y3).

To investigate the molecular mechanisms underlying the progressive reduction of yellow pigmentation in arils, RNA-Seq analysis was performed on ‘JJHN’ fruits at developmental stages Y2, Y3, Y4, and Y5. A total of 101.14 Gb clean reads from 12 libraries were obtained for downstream analysis, with each sample yielding at least 6 Gb clean data (**Supplementary Table S2**). Clean reads were mapped to the Feizixiao reference genome, with over 85% of reads uniquely aligned. Principal component analysis (PCA), based on the expression profiles of all genes, demonstrated tight clustering of biological replicates and clear separation among developmental stages (**Supplementary Figure S2**), confirming the reproducibility and biological relevance of the dataset. Differential expression analysis identified 8089 DEGs across the sampled stages (Dataset S3). Hierarchical clustering revealed distinct stage-specific expression patterns (**Figure 2A**), further supporting the dynamic transcriptional regulation during aril development. Pairwise comparisons identified 3,310 DEGs (1,712 upregulated) between Y2 and Y3, 1,593 DEGs (712 upregulated) between Y3 and Y4, and 2,657 DEGs (1,475 upregulated) between Y4 and Y5. The largest transcriptional shift occurred between Y2 and Y5, with 6,869 DEGs (3,487 upregulated) (**Figure 2B**). Each comparison group contained a substantial number of unique DEGs, while 150 DEGs were consistently differentially expressed across all stages (**Figure 2C**; Dataset S4). Notably, 4 genes associated with pigment biosynthesis, included *CHS1*, *DFR*, *ANS* (associated with flavonoid biosynthesis), and *ZEP.1*, (associated with carotenoid biosynthesis), were among the 150 conserved DEGs that persist across all stages.

GO enrichment analysis was performed for DEGs across developmental stages: Y3 vs Y2, Y4 vs Y3, and Y5 vs Y4 (**Figures 2D-F**). During the Y2–Y3 transition, several stress- and metabolism-related pathways were significantly upregulated, including cellular response to oxygen levels, pyruvate metabolic process, and regulation of protein sumoylation. Notably, genes associated with flavonoid biosynthetic process were significantly downregulated, consistent with metabolomic evidence showing a marked decline in flavonoid accumulation as the aril color began to fade. Between Y3 and Y4, the carotene catabolic process was significantly upregulated, corresponding to the observed reduction in carotenoid content during this stage. In the final stage from Y4 to Y5, genes involved in hydrogen peroxide metabolic process, positive regulation of reactive oxygen species metabolic process, and ethylene-activated signaling pathway were upregulated, suggesting increased oxidative activity and hormonal signaling during late-stage aril maturation.

Together, these findings highlight a coordinated transcriptional program involving the downregulation of flavonoid biosynthesis and upregulation of carotene degradation. These changes reflect the metabolic reprogramming and physiological transitions that accompany pigment loss and the maturation of ‘JJHN’ arils.

### 3.5 Transcriptome-metabolome coupled analysis reveals coordinated downregulation of flavonoid pathway genes during yellow pigment decline

**Figure 3:**
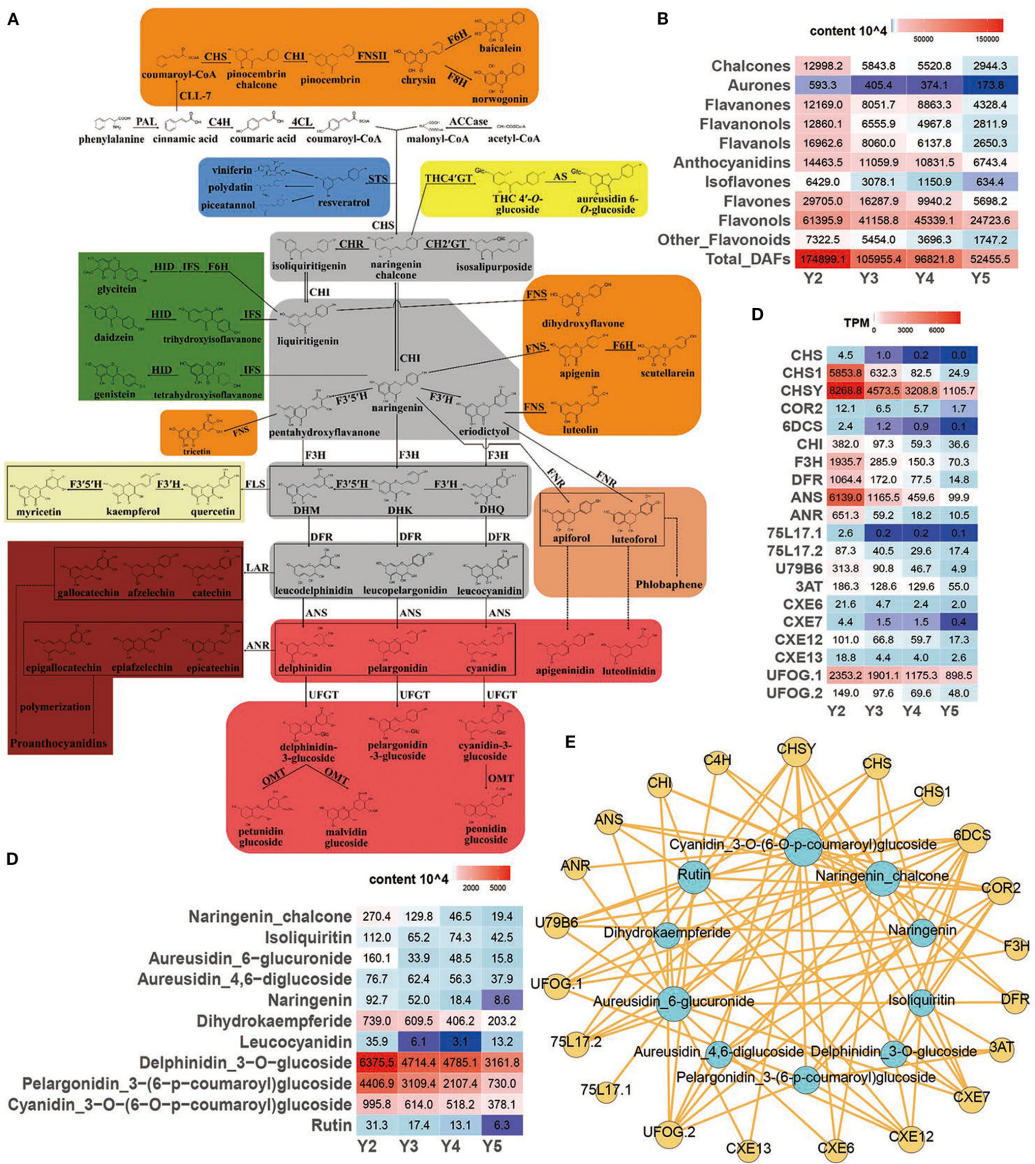
Regulation of flavonoid biosynthesis pathway in ‘JJHN’ arils. **(A)** Flavonoid pathway map contains eight branches (represented by the eight colored boxes) and four important intermediate metabolites (gray boxes). The enzyme names and flavonoid compounds are abbreviated as follows: PAL, phenylalanine ammonia lyase; C4H, cinnamic acid 4-hydroxylase; 4CL, 4-coumarate: CoA ligase; ACCase, acetyl-CoA carboxylase; STS, stilbene synthase; CHS, chalcone synthase; CHR, chalcone reductase; CH2´GT, chalcone 2´-glucosyltransferase; CH4´GT, chalcone 4´-O-glucosyltransferase; AS, aureusidin synthase; CHI, chalcone isomerase; FNS, flavone synthase; CLL-7, cinnamate–CoA ligase; F6H, flavonoid 6-hydroxylase; F8H, flavonoid 8-hydroxylase; IFS, isoflavone synthase; HID, 2-hydroxyisoflavanone dehydratase; FNR, flavanone 4-reductase; F3H, flavanone 3-hydroxylase; F3´5´H, flavanone 3´,5´-hydroxylase; DHK, dihydrokaempferol; DHQ, dihydroquercetin; DHM, dihydromyricetin; FLS, flavonol synthase; DFR, dihydroflavonol 4-reductase; ANS, anthocyanidin synthase; UFGT, UDP-glucose flavonoid 3-O-glucosyltransferase; OMT, O-methyl transferases; LAR, leucoanthocyanidin reductase; ANR, anthocyanidin reductase. **(B)** Relative quantification of flavonoid contents. **(C)** Expression levels (TPM) of key genes. **(D)** Relative quantification of key flavonoids. **(E)** Interomics correlation network of key metabolites and genes.

Transcriptome analysis revealed a coordinated downregulation of key structural genes involved in flavonoid biosynthesis from stages Y2 to Y5 in ‘JJHN’ arils, consistent with the observed decline in major flavonoid compounds, including chalcones, aurones, and anthocyanins (**Figures 3A–D**). Twenty representative DEGs associated with flavonoid biosynthesis exhibited decreasing expression trends (**Figure 3C**), indicating a progressive attenuation of this pathway during fruit development.

Within the flavonoid biosynthesis pathway (ko00941), chalcone synthase genes (*CHS*, *CHS1*, *CHSY*)—catalyzing the first committed step—were markedly downregulated, while chalcone reductases (*COR2*, *6DCS*) downregulated as well, suggesting a reduction in chalcone-derived pigment production. Downstream gene chalcone isomerase (*CHI*), which converts chalcones to flavanones, also showed declining expression, reflecting reduced upstream metabolic flux. Further downstream, genes such as *F3H* (flavanone 3-hydroxylase) and *DFR* (dihydroflavonol 4-reductase)—key enzymes in the biosynthesis of flavanonols and flavonols—exhibited similarly reduced transcript levels. Anthocyanin-modifying genes, including *ANS* (anthocyanidin synthase), *ANR* (anthocyanidin reductase), *UFGT*s (glycosyltransferases), and *3AT* (acyltransferases), were also downregulated, suggesting limited anthocyanin accumulation at later developmental stages. Genes related to isoflavonoid biosynthesis (*CXE6*, *CXE7*, *CXE12*, *CXE13*) and flavones/flavonols production (*UFOG.1*, *UFOG.2*) were similarly repressed, indicating broad suppression across multiple flavonoid sub-branches.

Most genes peaked in expression at the early developmental stage (Y2), correlating with the accumulation of flavonoid pigments, particularly yellow pigment-associated compounds such as chalcones and aurones (**Figures 3B, C**). Co-expression network analysis integrating DEGs and DAFs (**Figure 3E**) revealed strong gene–metabolite correlations, underscoring the role of transcriptional regulation in modulating pathway flux and metabolite accumulation. Collectively, these results suggest that transcriptional repression of the flavonoid biosynthetic pathway contributes to the decreased pigment content and fading yellow coloration in ‘JJHN’ arils during fruit maturation.

### 3.6 Reduced carotenoid biosynthesis contributed to yellow color attenuation

**Figure 4:**
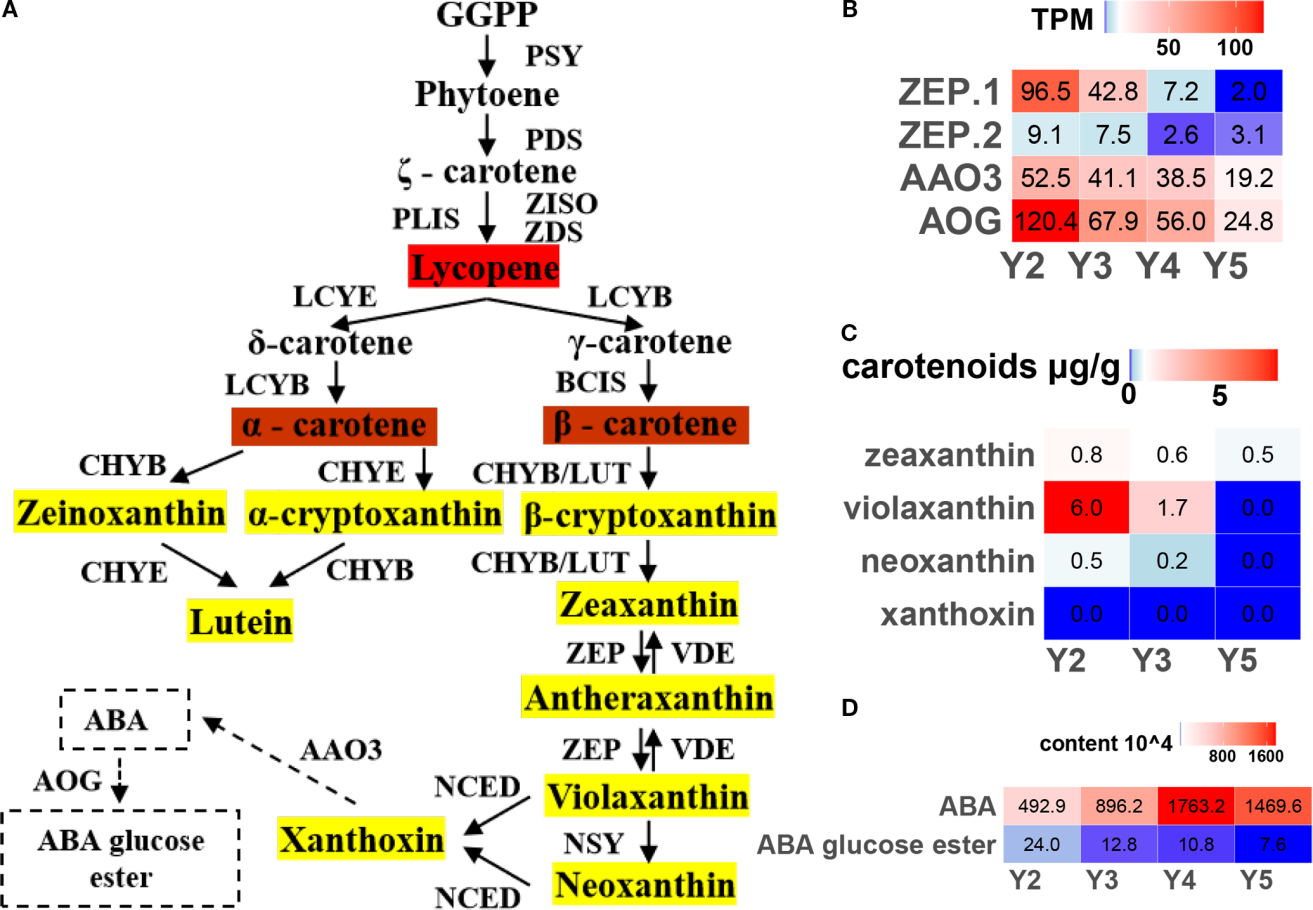
Regulation of carotenoid biosynthesis pathway in ‘JJHN’ arils. **(A)** The pathway uses GGPP geranyl-geranyl-PP to phytoene to convert it into downstream products. The enzymes catalyzing each step in the pathways are as following: PSY, phytoene synthases; PDS, phytoene desaturase; ZISO, ζ-carotene isomerase; ZDS, ζ-carotene desaturase; PLIS, prolycopene isomerases; LCYE, Lycopene ξ-cyclase; LCYB, lycopene β-cyclase; BCIS, β-carotene isomerases; CHYB/E, ζ/β -ring hydroxlases (or LUT5); ZEP, zeaxanthin epoxidase (or ABA1); VDE, violaxanthin de-epoxidase; NSY, neoxanthin synthase; NCED, 9-cis-epoxycarotenoid dioxygenase; AAO3, abscisic-aldehyde oxidase; and AOG, abscisate beta-glucosyltransferase. **(B)** Expression levels (TPM) of key genes. **(C)** Absolute quantification of carotenoids. **(D)** Relative quantification of ABA.

In the carotenoid biosynthesis pathway (**Figure 4A**), two *ZEP* genes (zeaxanthin epoxidase), which catalyze the conversion of zeaxanthin to violaxanthin, showed peak expression at the early developmental stage (Y2), followed by a clear decline as the fruit matured (**Figure 4B**). Consistently, violaxanthin content, as determined by carotenoid-targeted metabolomics, also decreased over time (**Figure 4C**). The decline in violaxanthin biosynthesis, together with the weakened carotenoid flux, likely contributes to the gradual fading of yellow pigmentation in ‘JJHN’ arils.

As violaxanthin is a direct precursor of abscisic acid (ABA), we further examined the expression of ABA-related genes and the corresponding metabolite levels. Although both the key ABA biosynthesis gene *AAO3* and the catabolic gene *AOG* were downregulated during fruit development (**Figure 4B**), widely targeted metabolomics revealed an increase in free ABA levels, accompanied by a reduction in its major storage form, ABA-glucose ester (ABA-GE) (**Figure 4D**). These findings suggest that post-transcriptional or post-translational mechanisms may govern the dynamic balance between violaxanthin-to-ABA conversion and ABA degradation in ‘JJHN’ arils during fruit development.

### 3.7 Network analysis and validation candidate genes associated with pigment metabolism

**Figure 5:**
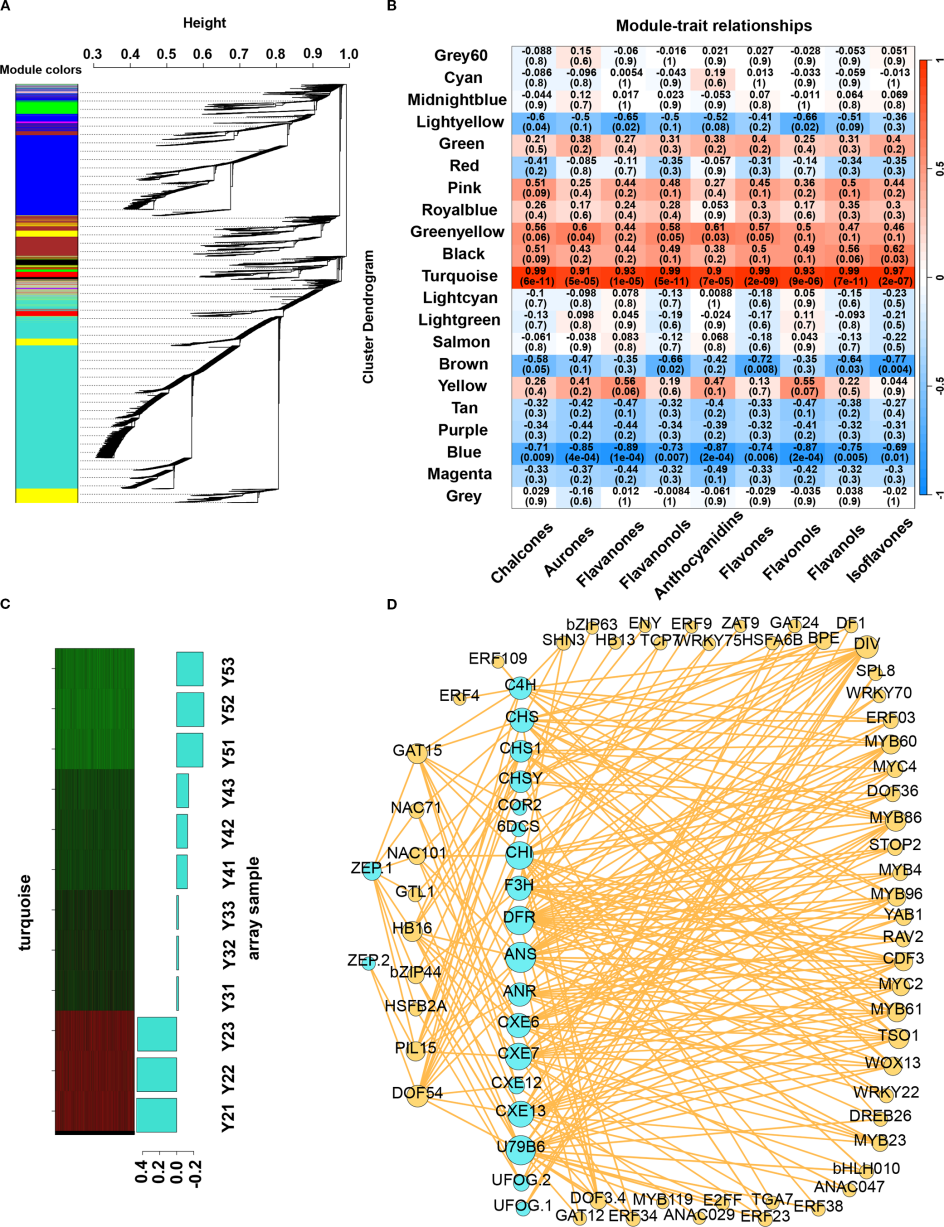
WGCNA for candidate gene screening. **(A)** Transcript clustering and module identification. The different colors represent different modules. **(B)** Correlation between modules and flavonoid contents. **(C)** The eigengenes expression patterns and gene expression heatmap across samples for the “turquoise” module. **(D)** Network diagram of correlations between structure genes and TFs in TFBS.

To identify TF–gene targets involved in flavonoid and carotenoid metabolism during the yellow fading of litchi arils, WGCNA was conducted using expression data from 17,215 genes. A hierarchical clustering dendrogram based on the topological overlap matrix (TOM) was generated, grouping genes with similar expression profiles into 21 co-expression modules (**Figure 5A**). Among these, the “turquoise” module showed the strongest positive correlation with flavonoid content in ‘JJHN’ arils during fruit development (**Figures 5B-C**), and was therefore selected for further analysis. This module contained 6,956 genes, from which an undirected weighted network with scale-free topology was constructed.

Network analysis in Cytoscape identified 346 hub genes within the top 5% of connectivity (Dataset S5). These included two hub genes—*CHSY* (*LITCHI020852*) was involved in flavonoid biosynthesis (ko00941), and *U79B6* (*LITCHI013091*) participated in anthocyanin biosynthesis (ko00942)—and 9 hub TFs, included *DIV*, *PIL15*, *NAC71*, *GAT15*, *DOF54*, *TCP7*, *MYC2*, *MYB86*, *GAT24*. Notably, the 9 hub TFs are components of the MYB–bHLH–WD40 (MBW) complex, a key regulatory module that controls flavonoid and carotenoid biosynthesis.

Genes involved in flavonoid and carotenoid metabolism, along with their co-expressed TFs, were identified from the “turquoise” module. To enhance network reliability and minimize false-positive associations, a weight threshold of >0.4 was applied. Furthermore, only TF–target gene interactions supported by the presence of corresponding transcription factor binding sites (TFBS) were retained to improve the specificity of the regulatory network (**Figure 5D**, Dataset S6). A total of 57 TFs (**Supplementary Table S3**) were identified, along with their corresponding DNA-binding motifs. Among these, 48 TFs—predominantly from families such as bHLH, bZIP, MYB, ERF, and DOF—exhibited strong positive correlations with structural genes involved in flavonoid and carotenoid biosynthesis. For example, *F3H* and *bZIP44* showed coordinated downregulation during the ripening of ‘JJHN’, suggesting a role in pigment accumulation. Notably, 24 of these TFs were predicted to co-regulate both flavonoid and carotenoid metabolic pathways, underscoring the interconnectedness of these two pigment biosynthesis networks. In contrast, 10 TFs were negatively associated with flavonoid biosynthesis. For instance, homologs of *ANAC029* may bind to the promoter region of 2-hydroxyisoflavanone dehydratase (*HID*), thereby repressing its expression. Similarly, two WRKY family members were predicted to negatively regulate *CHI* and *DFR*, while homologs of *bZIP63*, *DF1*, and *GTL1* were negatively associated with the expression of *CHS*. Overall, several structural genes—including *C4H*, *CHS*, *CHI*, *F3H* and *ZEP*—exhibited co-expression with multiple transcription factors. This highlights a tightly coordinated and intricate transcriptional network regulating flavonoid and carotenoid metabolism during fruit development and ripening in litchi.

### 3.8 qRT-PCR validation of RNA-seq data

**Figure 6:**
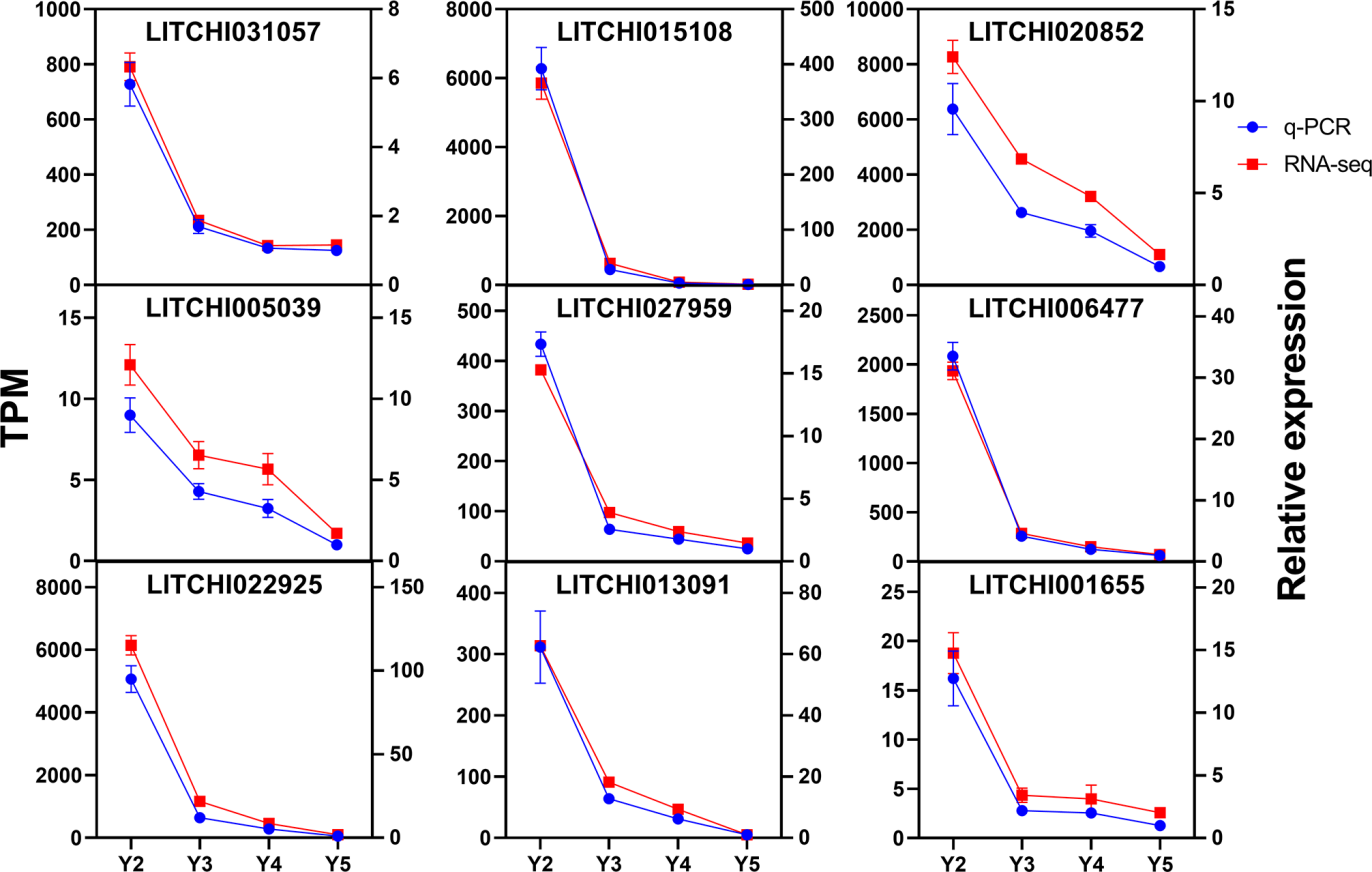
Expression patterns of nine flavonoid-related genes in ‘JJHN’ arils at five developmental stages. Vertical bars represent mean values ± standard error (SE) from three independent biological replicates.

To assess the reliability of the transcriptome sequencing results in ‘JJHN’ litchi arils, nine flavonoid-related genes were selected for validation using quantitative real-time PCR (qRT-PCR) with gene-specific primers (**Supplementary Table S4**). The qRT-PCR analysis showed that the relative expression patterns of these genes closely mirrored the trends obtained from RNA-Seq (**Figure 6**). These results confirm the robustness of the RNA-Seq data and supports its suitability for investigating differential gene expression in this study.

## 4 Discussion

### 4.1 Coordinated decline of flavonoids and carotenoids orchestrated attenuation of yellow coloration in developing ‘JJHN’ litchi arils

Litchi is widely appreciated for its distinctive flavor and crimson pericarp, with previous studies attributing its red pericarp coloration to the accumulation of cyanidin 3-rutinoside (Xu et al., 2024). In contrast, the arils of most litchi cultivars are white or creamy white due to the lack of visible pigments, despite containing abundant colorless flavonoids and low carotenoid levels (Su et al., 2014; Septembre-Malaterre et al., 2016). Both flavonoids and carotenoids are known contributors to fruit pigmentation, influencing a spectrum of colors from yellow to red in many horticultural species (Su et al., 2023; Zhou et al., 2024). The ‘JJHN’ cultivar is notable for its distinctive aril color transition from orange-yellow to pale yellow during development. Quantification of TAF and TLC in ‘JJHN’ arils confirmed the activation of both biosynthetic pathways, implicating both pigment classes in aril coloration.

Widely targeted metabolomics identified flavonoid subclasses—including aurones, chalcones, flavones, and flavonols—with peak accumulation at the early developmental stage (Y2), coinciding with the orange-yellow hue of the arils. Aurones and chalcones, known for their yellow pigmentation (Vicente and Boscaiu, 2018; Liu et al., 2021), are likely primary contributors to this coloration. Concurrently, violaxanthin, the dominant carotenoid comprising 62.4% of total carotenoids at Y2, also peaked at this stage but declined by 72.2% at Y3 and became undetectable at maturity (detection sensitivity: > 10 ng/g). This temporal pattern resembles violaxanthin-driven yellow pigmentation reported in other species such as potato (Zhang et al., 2023).

The parallel decline of yellow-associated flavonoids (particularly aurones and chalcones) and violaxanthin from Y2 to Y5 corresponds with the gradual fading of yellow coloration. Notably, a slight increase in select flavonoid compounds—including aureusidin 6-glucuronide, isobavachalcone glucoside, naringin chalcone, isoliquiritin, and marein—between Y3 and Y4 may underlie a brief intensification of yellow tone during mid-development, while flavones and flavonols, which exhibit limited chromogenic potential, likely contribute to subtle modulation of aril hue rather than visible coloration (Liu et al., 2021). Anthocyanins such as cyanidin 3-O-rutinoside and peonidin 3-O-rutinoside were detected in varying degrees of accumulation, which could not be seen with the naked eye as red pigmentation in the ‘JJHN’ aril, may likely due to their low absolute contents (Bhoopat et al., 2011).

In summary, these findings support a dual-pigment model in which the dynamic interplay between carotenoids and flavonoids collectively governs the development and subsequent fading of yellow coloration in ‘JJHN’ arils. This work deepens our understanding of pigment interactions in litchi and highlights the importance of coordinated regulation between pigment biosynthetic pathways in determining fruit color phenotype.

### 4.2 Transcriptional coordination of flavonoid and carotenoid biosynthesis underlay stage-specific pigmentation in ‘JJHN’ litchi arils

The molecular mechanisms underlying pigment accumulation in ‘JJHN’ arils involve coordinated regulation of structural genes and transcription factors governing both flavonoid and carotenoid biosynthesis. Previous studies have established that flavonoid production is regulated by the structural genes including *PAL*, *C4H*, *CHS*, *CHI*, *F3H*, *F3’H*, *DFR*, *ANS*, *UFGT*, and *GST*, with differential expression patterns correlating with pigment accumulation across various species (Vicente and Boscaiu, 2018). In the present study, DEGs associated with flavonoid biosynthesis—including 3 *CHS*s, 2 *CHR*s, *CHI*, *DFR*, *ANS*, *ANR*, 3 *UFGT*s, *3AT*s, 4 *HIDH*s, and 2 *FG2*s—exhibited a consistent downward trend from Y2 to Y5, corresponding to the observed decline in flavonoid content and yellow coloration intensity.

Transcriptional regulation represents a critical control point in pigment biosynthesis, with the MBW complex serving as the primary regulatory mechanism for flavonoid production (Xu et al., 2015). Our transcriptomic analysis identified multiple transcription factor families—including bHLH, bZIP, MYB, ERF, and DOF—that exhibited strong positive correlations with structural genes involved in both flavonoid and carotenoid biosynthesis. Notably, *F3H* and *bZIP44* demonstrated coordinated downregulation during ‘JJHN’ ripening, with potential DNA-binding motifs identified between these factors. This regulatory interaction aligns with previous findings in pear, where *PpbZIP44* directly binds to *PpF3H* promoters to enhance flavonoid accumulation (Wang et al., 2023). Conversely, transcription factors including *NACs*, *WRKYs*, and additional *bZIPs* showed negative associations with flavonoid biosynthesis. Two WRKY family members were predicted to negatively regulate *CHI* and *DFR* expression, consistent with established roles of WRKY proteins as negative regulators of anthocyanin biosynthesis through direct promoter binding and interference with MBW complex function (Tao et al., 2024).

The regulation of carotenoid biosynthesis in ‘JJHN’ arils involves *ZEP*, which catalyzes the conversion of zeaxanthin to violaxanthin while simultaneously initiating ABA biosynthesis (Liu et al., 2020; Zhang et al., 2023). The downregulation of *ZEP* genes from Y2 to Y5 likely underlies the dramatic decline in violaxanthin content, which decreased by 72.2% from Y2 to Y3 and became undetectable at maturity. Interestingly, despite the downregulation of the ABA biosynthesis gene *AAO3* and the ABA catabolism gene *AOG*, ABA content increased in litchi arils during development. This paradoxical pattern suggests that ABA accumulation results from reduced catabolism exceeding the diminished biosynthetic rate, reflecting the complex regulatory balance governing ABA homeostasis during fruit ripening (Xu et al., 2002; Zhang and Gan, 2012; Yang et al., 2014).

Our analysis identified 48 TFs, predominantly from bHLH, bZIP, MYB, ERF, and DOF families, that exhibited binding potential with structural genes involved in both flavonoid and carotenoid biosynthesis. This regulatory overlap suggests potential crosstalk between pigment biosynthetic pathways, similar to observations in tomato where *SlMYB72* simultaneously regulates carotenoid genes (*PSY*, *ZISO*, *LCYB*) and flavonoid genes (*4CL*, *CHS*) (Wu et al., 2020). The coordinated regulation of both pigment classes in ‘JJHN’ arils supports the dual-pigment model proposed earlier and provides mechanistic insight into the synchronized decline of yellow-associated compounds during fruit development.

These findings collectively demonstrate that the unique color phenotype of ‘JJHN’ arils results from the coordinated transcriptional control of multiple pigment biosynthetic pathways, with the temporal decline in key regulatory factors driving the characteristic transition from orange-yellow to pale yellow coloration during fruit maturation.

## 5 Conclusion

This study revealed the metabolic and genetic mechanisms behind the yellow aril coloration and its fading during fruit development in the *Litchi chinensis* cv. ‘JJHN’. Integrated metabolomic and transcriptomic analyses showed that early-stage yellow pigmentation was primarily due to the accumulation of aurones, chalcones, and violaxanthin. As the fruit matures, the levels of these pigments decreased significantly, resulting in the gradual loss of yellow color. This pigment decline was closely linked to the downregulation of key biosynthetic genes in the flavonoid and carotenoid pathways, including *CHS*, *CHR*, *CHI*, *ZEP* and *AAO3*. Co-expression network analysis identified transcription factors—mainly from the MYB, bHLH, bZIP, NAC, and DOF families—that were likely involved in regulating these genes. Many of these transcription factors were predicted to co-regulate both pigment pathways, highlighting the coordinated control of flavonoid and carotenoid metabolism. Overall, our findings supported a dual-pigment model for yellow coloration in ‘JJHN’ arils and demonstrate that the color shift from orange-yellow to pale yellow was regulated by transcriptional suppression of pigment biosynthesis. These results provided new insights into fruit color regulation and offer molecular targets for breeding litchi cultivars with improved aril pigmentation.

## Data Availability

The original contributions presented in the study are publicly available. The RNA-seq data have been deposited in the National Genomics Data Center (https://ngdc.cncb.ac.cn/), BioProject accession PRJCA043210.
