# Supplementary material for: Multi-omics elucidation of yellow aril coloration in litchi (Litchi chinensis Sonn.) cultivar ‘Jianjianghongnuo’: coordinated downregulation of flavonoid and carotenoid biosynthetic pathways drives pigment dynamics
Source: Front Plant Sci. 2025 Oct 6;16:1669458. doi: 10.3389/fpls.2025.1669458 (PMC12535983; doi:10.3389/fpls.2025.1669458)
Supplement: Supplementary file 1 [file DataSheet1.zip › 250926Re-submit Supplementary Material/Supplementary Methods S1 Analytical conditions of widely targeted metabolome and quantitative identification of metabolites.docx]

Supplemental Methods S1

Analytical conditions of widely targeted metabolome and quantitative identification of metabolites.

Remove the samples from the -80 ℃ refrigerator, thaw, and vortex for 30 S to mix. Take 9 mL of the sample, place it in the corresponding numbered 50 mL centrifuge tube, freeze it in the -80 ℃ refrigerator overnight, and vacuum freeze dry. After freeze-drying, add 70% methanolic internal standard extract at a ratio of 30 times the concentration, e.g. add 300 μL extractant to 9 mL samples after freeze-drying and 200 μL extractant to 6 mL samples after freeze-drying. Vortex for 15 min and sonicate in an ice water bath (KQ5200E) for 10 min. 12,000 r/min and centrifuge at 4°C (5424R , Eppendorf) for 3 min. The supernatant was removed, filtered through a microporous membrane (0.22 μm pore size), and stored in the injection vial for LC-MS/MS detection.

The sample extracts were analyzed using an UPLC-ESI-MS/MS system (UPLC, ExionLC™ AD ， https://sciex.com.cn/) and Tandem mass spectrometry system (https://sciex.com.cn/). The analytical conditions were as follows, UPLC: column, Agilent SB-C18 (1.8 µm, 2.1 mm * 100 mm); The mobile phase was consisted of solvent A, pure water with 0.1% formic acid, and solvent B,cetonitrile with 0.1% formic acid. Sample measurements were performed with a gradient program that employed the starting conditions of 95% A, 5% B.Within 9 min, a linear gradient to 5% A, 95% B was programmed, and a composition of 5% A, 95% B was kept for 1 min. Subsequently, a composition of 95% A, 5.0% B was adjusted within 1.1 min and kept for 2.9 min. The flowvelocity was set as 0.35 mL per minute; The column oven was set to 40°C; The injection volume was 2 μL. The effluent was alternatively connected to an ESI-triple quadrupolelinear ion trap (QTRAP)-MS.

The ESI source operation parameters were as follows: source temperature 500°C; ion spray voltage (IS) 5500 V (positive ion mode)/-4500 V (negative ion mode); ion source gas I (GSI), gas II(GSII), curtain gas (CUR) were set at 50, 60, and 25 psi, respectively; the collision-activated dissociation(CAD) was high. QQQ scans were acquired as MRM experiments with collision gas (nitrogen) set to medium. DP(declustering potential) and CE(collision energy) for individual MRM transitions was done with further DP and CE optimization. A specific set of MRM transitions were monitored for each period according to the metabolites eluted within this period.
