# Supplementary material for: Multi-omics elucidation of yellow aril coloration in litchi (Litchi chinensis Sonn.) cultivar ‘Jianjianghongnuo’: coordinated downregulation of flavonoid and carotenoid biosynthetic pathways drives pigment dynamics
Source: Front Plant Sci. 2025 Oct 6;16:1669458. doi: 10.3389/fpls.2025.1669458 (PMC12535983; doi:10.3389/fpls.2025.1669458)
Supplement: Supplementary file 1 [file DataSheet1.zip › 250926Re-submit Supplementary Material/Supplementary Methods S2 Analytical conditions of Targeted metabolomics analysis of carotenoids and quantitative identification of.docx]

Supplemental Methods S2

Analytical conditions of Targeted metabolomics analysis of carotenoids and quantitative identification of metabolites.

The sample extracts were analyzed by an UPLC-MS/MS system (UPLC, Exion LC™ AD; MS/MS, QTRAP® 6500+). The UPLC conditions were as follow, UPLC: chromatographic column, YMC C30; Mobile phase, methanol and acetonitrile mixture (methanol : acetonitrile = 1 : 3, v/v) with 0.01% BHT and 0.1% formic acid (phase A), methyl tert-butyl ether with 0.01% BHT (phase B); gradient elution program, The volume ratios of phase A and phase B were 100 : 0, 100 : 0, 30 : 70, 5 : 95, 100 : 0 and 100 : 0 at 0 min, 3 min, 5 min, 9 min, 10 min and 11 min, respectively; flow rate, 0.8 mL/min; column temperature, 28 °C; injection volume, 2 μL. MS/MS conditions: ion source, APCI; source temperature, 350 °C; curtain gas, 25.0 psi. Based on the optimized de-clustering potential and collision energy, each ion pair was scanned and detected in Q-Trap 6500+. In addition, a quality control (QC) sample mixed with standards was inserted for every 10 test samples during instrument analysis to judge the stability of the instrument during project detection.

Qualitative analysis of the mass spectrometry data was based on the MWDB database (Metware Biotechnology Co., Ltd.) established by standards. The MRM model of QQQ mass spectrometry was used for the quantitative analysis of carotenoids. In addition, solutions at different concentrations (0.01 μg/mL, 0.05 μg/mL, 0.1 μg/mL, 0.5 μg/mL, 1 μg/mL, 5 μg/mL, 10 μg/mL, and 40 μg/mL) were prepared for all carotenoid standards to draw the standard curves of different carotenoid standards (Supporting Information, File 1). At last, the area ratios of each chromatographic peak of all the samples and the internal standard peak were substituted into the standard curve equation of the corresponding standard substance, and the absolute content data of the substance in the samples to be tested were calculated. The mass spectrum data of all the samples were analyzed using Analyst 1.6.3 software.
