# Supplementary material for: Multi-omics elucidation of yellow aril coloration in litchi (Litchi chinensis Sonn.) cultivar ‘Jianjianghongnuo’: coordinated downregulation of flavonoid and carotenoid biosynthetic pathways drives pigment dynamics
Source: Front Plant Sci. 2025 Oct 6;16:1669458. doi: 10.3389/fpls.2025.1669458 (PMC12535983; doi:10.3389/fpls.2025.1669458)
Supplement: Supplementary file 1 [file DataSheet1.zip › 250926Re-submit Supplementary Material/Supplementary Table S1 Correlation analysis between the aurones chalcones content and b values.docx]

Table S1. Correlation analysis between the aurones, chalcones content and b values

| **CIE** | **Compounds** | **Class** | **R** |
| --- | --- | --- | --- |
| **b*** | **Aurones DAMs** | **Aurones** | **0.861443599** |
| **b*** | **Chalcones DAMs** | **Chalcones** | **0.807951969** |
| b* | Aureusidin 6-glucuronide | Aurones | 0.763246905 |
| b* | Cernuoside* | Aurones | 0.748808403 |
| b* | Maesopsin 6-beta-D-glucopyranoside | Aurones | 0.68354402 |
| b* | Aureusidin 4,6-diglucoside | Aurones | 0.857990514 |
| b* | Xanthohumol | Chalcones | 0.770180196 |
| b* | Phlorizin chalcone | Chalcones | 0.85532991 |
| b* | Phloretin-2'-O-(6''-O-acetyl)glucoside | Chalcones | 0.628562076 |
| b* | Sieboldin | Chalcones | 0.743597349 |
| b* | Hydroxy isoliquiritigenin glucoside* | Chalcones | 0.751327248 |
| b* | Naringenin chalcone | Chalcones | 0.724606728 |
| b* | Dihydrocharcone-4'-O-glucoside* | Chalcones | 0.772431406 |
| b* | Phlorizin | Chalcones | 0.652505921 |
| b* | Phloretin-4'-O-glucoside (Trilobatin)* | Chalcones | 0.918640553 |
| b* | Neosakuranin | Chalcones | 0.827319936 |
| b* | Dihydromarein | Chalcones | 0.809459374 |
| b* | Phloretin-2'-O-(6''-O-xylosyl)glucoside | Chalcones | 0.821213988 |
| b* | Pterosupin | Chalcones | 0.747871903 |
| b* | Phloretin-2'-O-(6''-O-rhamnoside)glucoside | Chalcones | 0.694925294 |
| b* | Marein | Chalcones | 0.378612237 |
| b* | 3-Hydroxyphloretin-4'-O-glucoside | Chalcones | 0.736445288 |
| b* | Isobavachalcone glucoside | Chalcones | 0.745044313 |
| b* | Naringin chalcone* | Chalcones | -0.490688463 |
| b* | 2',3,4,4',6'-Peptahydroxychalcone 4'-O-glucoside | Chalcones | 0.743499111 |
| b* | Isoliquiritin | Chalcones | 0.70864491 |
| b* | Homobutein 4-glucoside | Chalcones | 0.850749868 |
| b* | 4-Hydroxychalcone | Chalcones | 0.74784721 |
| b* | Homoeriodictyolchalcone 2'-glucoside* | Chalcones | 0.752727327 |
| b* | Okanin-4'-(6''-O-acetyl)glucoside* | Chalcones | 0.674654768 |
| b* | 3-HydroxyPhloretin-2'-O-(6''-O-xylosyl)glucoside | Chalcones | -0.485685548 |
