# Supplementary material for: Multi-omics elucidation of yellow aril coloration in litchi (Litchi chinensis Sonn.) cultivar ‘Jianjianghongnuo’: coordinated downregulation of flavonoid and carotenoid biosynthetic pathways drives pigment dynamics
Source: Front Plant Sci. 2025 Oct 6;16:1669458. doi: 10.3389/fpls.2025.1669458 (PMC12535983; doi:10.3389/fpls.2025.1669458)
Supplement: Supplementary file 1 [file DataSheet1.zip › 250926Re-submit Supplementary Material/Supplementary Table S2 Quality statistics of transcriptome sequencing data.docx]

Table S2. Quality statistics of transcriptome sequencing data

| Sample | Group | Raw Reads | Clean  Reads | Clean  Base(G) | Error Rate (%) | Q20 (%) | Q30 (%) | GC  Content (%) |
| --- | --- | --- | --- | --- | --- | --- | --- | --- |
| Y21 | Y2 | 58637018 | 57719754 | 8.66 | 0.01 | 98.45 | 95.34 | 44.4 |
| Y22 | Y2 | 62093558 | 61177024 | 9.18 | 0.01 | 98.48 | 95.45 | 44.67 |
| Y23 | Y2 | 50838520 | 50013590 | 7.5 | 0.01 | 98.52 | 95.47 | 44.54 |
| Y31 | Y3 | 51167316 | 50270518 | 7.54 | 0.01 | 98.43 | 95.3 | 44.72 |
| Y32 | Y3 | 56055010 | 55113772 | 8.27 | 0.01 | 98.49 | 95.46 | 44.75 |
| Y33 | Y3 | 65914838 | 64763408 | 9.71 | 0.01 | 98.5 | 95.51 | 44.62 |
| Y41 | Y4 | 68799838 | 67559000 | 10.13 | 0.01 | 98.57 | 95.66 | 44.75 |
| Y42 | Y4 | 57095526 | 55995934 | 8.4 | 0.01 | 98.51 | 95.57 | 44.63 |
| Y43 | Y4 | 43547354 | 42781092 | 6.42 | 0.01 | 98.43 | 95.3 | 44.73 |
| Y51 | Y5 | 49949144 | 49002156 | 7.35 | 0.01 | 98.47 | 95.4 | 44.75 |
| Y52 | Y5 | 55252898 | 54285372 | 8.14 | 0.01 | 98.45 | 95.35 | 45.26 |
| Y53 | Y5 | 66818020 | 65607500 | 9.84 | 0.01 | 98.47 | 95.45 | 44.85 |
