# Supplementary material for: Multi-omics elucidation of yellow aril coloration in litchi (Litchi chinensis Sonn.) cultivar ‘Jianjianghongnuo’: coordinated downregulation of flavonoid and carotenoid biosynthetic pathways drives pigment dynamics
Source: Front Plant Sci. 2025 Oct 6;16:1669458. doi: 10.3389/fpls.2025.1669458 (PMC12535983; doi:10.3389/fpls.2025.1669458)
Supplement: Supplementary file 1 [file DataSheet1.zip › 250926Re-submit Supplementary Material/Supplementary Table S3 57 TFs of the regulatory network by TFBS.docx]

Table S3. 57 TFs of the regulatory network by TFBS

| ID | GN | Y2 | Y3 | Y4 | Y5 |
| --- | --- | --- | --- | --- | --- |
| LITCHI001614 | ERF109 | 4.3 | 0.8 | 1.2 | 1.7 |
| LITCHI001854 | bZIP44 | 556.3 | 262.4 | 341.5 | 206.2 |
| LITCHI002019 | MYB4 | 15 | 10.5 | 10.5 | 8.9 |
| LITCHI002253 | MYB61 | 8.8 | 1.1 | 0.6 | 0.3 |
| LITCHI003293 | MYB60 | 6.3 | 0.7 | 0.4 | 0 |
| LITCHI003437 | ANAC047 | 19.4 | 94 | 89.1 | 82.9 |
| LITCHI003634 | bZIP63 | 0.4 | 0.8 | 1.4 | 2 |
| LITCHI003672 | TSO1 | 14 | 7.9 | 5.7 | 4.8 |
| LITCHI004628 | WRKY75 | 22.6 | 89.3 | 65 | 95 |
| LITCHI005070 | BPE | 8.9 | 3.2 | 0.8 | 0.1 |
| LITCHI005143 | HB13 | 87.1 | 50.9 | 54.7 | 17.5 |
| LITCHI005204 | ANAC029 | 85.3 | 323 | 526.4 | 747.7 |
| LITCHI005481 | RAV2 | 32.8 | 77.7 | 69.1 | 63 |
| LITCHI005896 | TCP7 | 136.5 | 81.6 | 67.6 | 48.2 |
| LITCHI006756 | DOF3.4 | 16.4 | 1 | 0.6 | 0.3 |
| LITCHI007634 | DF1 | 22.8 | 41 | 80.2 | 96.9 |
| LITCHI008829 | NAC101 | 0.9 | 0.2 | 0.1 | 0.1 |
| LITCHI009042 | ERF34 | 1.7 | 0.2 | 0 | 0.1 |
| LITCHI009313 | HSFB2A | 48.1 | 64.1 | 69.9 | 73.9 |
| LITCHI009957 | WOX13 | 1.6 | 0.2 | 0.2 | 0.1 |
| LITCHI010247 | bZIP44 | 240.1 | 91.4 | 22.5 | 14.3 |
| LITCHI010336 | ENY | 22.3 | 14.7 | 13 | 4.5 |
| LITCHI010386 | TGA7 | 32.9 | 17.1 | 19.5 | 8.9 |
| LITCHI010571 | HB16 | 44.9 | 8 | 6.5 | 0.1 |
| LITCHI010692 | TIFY1 | 98.4 | 70.8 | 66.6 | 56.5 |
| LITCHI011225 | DREB26 | 36.5 | 15 | 1.3 | 0.3 |
| LITCHI011739 | bHLH010 | 4.1 | 0.6 | 0.7 | 0.6 |
| LITCHI013074 | ERF4 | 23.1 | 11.7 | 10.3 | 6.8 |
| LITCHI014411 | GA15 | 31.2 | 15.7 | 11.5 | 5.1 |
| LITCHI014879 | HSFA6B | 1.2 | 0.8 | 0.5 | 0.1 |
| LITCHI016997 | SHN3 | 2 | 0.4 | 0.2 | 0.1 |
| LITCHI017192 | GA12 | 1.3 | 0.4 | 0 | 0.4 |
| LITCHI017547 | GRF6 | 4.5 | 0.6 | 0.1 | 0.6 |
| LITCHI018253 | DIV | 424.3 | 177.1 | 136.6 | 36.2 |
| LITCHI019609 | WRKY70 | 2.1 | 0.7 | 0.7 | 0.2 |
| LITCHI020020 | GTL1 | 29 | 55.3 | 76.9 | 91.3 |
| LITCHI020398 | NAC71 | 108.7 | 55.2 | 49.8 | 21.4 |
| LITCHI020533 | MYB23 | 3.1 | 0.1 | 0 | 0 |
| LITCHI021833 | MYC4 | 1.6 | 0.3 | 0.4 | 0 |
| LITCHI021899 | ERF23 | 11.8 | 0.5 | 0 | 0 |
| LITCHI022288 | DEL1 | 66.3 | 17.2 | 13.5 | 4.5 |
| LITCHI022764 | ERF38 | 2 | 0.8 | 0.3 | 0 |
| LITCHI022787 | ZAT9 | 4.6 | 0.6 | 0.1 | 0.1 |
| LITCHI023309 | DOF5.4 | 14 | 7.5 | 5.2 | 2.8 |
| LITCHI023435 | MYB31 | 23.7 | 7.4 | 7 | 6.3 |
| LITCHI023451 | CDF3 | 12 | 3.2 | 3.1 | 4.4 |
| LITCHI024576 | STOP2 | 0.5 | 1.6 | 1.5 | 1.6 |
| LITCHI025088 | MYB119 | 6.7 | 3.1 | 1.9 | 0.8 |
| LITCHI025415 | SPL8 | 13.4 | 7.3 | 4.4 | 1.4 |
| LITCHI025506 | AFO | 3 | 0.3 | 0.2 | 0 |
| LITCHI025778 | WRKY22 | 6.8 | 28 | 22 | 25.9 |
| LITCHI026700 | PAP3 | 22 | 10.1 | 6 | 0.5 |
| LITCHI026809 | MYC4 | 12 | 3.2 | 1.6 | 0 |
| LITCHI027743 | OBP3 | 3.2 | 1 | 0.7 | 0.3 |
| LITCHI028489 | MYB67 | 39.2 | 12.5 | 12.2 | 3.2 |
| LITCHI029145 | ERF9 | 71 | 9.8 | 35.6 | 19.7 |
| LITCHI030961 | ESE3 | 56.7 | 2.8 | 0.1 | 0.1 |
