# Supplementary material for: Multi-omics elucidation of yellow aril coloration in litchi (Litchi chinensis Sonn.) cultivar ‘Jianjianghongnuo’: coordinated downregulation of flavonoid and carotenoid biosynthetic pathways drives pigment dynamics
Source: Front Plant Sci. 2025 Oct 6;16:1669458. doi: 10.3389/fpls.2025.1669458 (PMC12535983; doi:10.3389/fpls.2025.1669458)
Supplement: Supplementary file 1 [file DataSheet1.zip › 250926Re-submit Supplementary Material/Supplementary Table S4 Primers for qPCR.docx]

Table S4. Primers for qPCR

| Gene ID | Primer ID | Primer Sequence | Primer Tm | Product Size |
| --- | --- | --- | --- | --- |
| LITCHI001655 | q01655F | AACCGATCCCAGGCGAGA | 60.364 | 150 |
| LITCHI001655 | q01655R | TGGTGCATCCAAGCCGAG | 60.046 | 150 |
| LITCHI005039 | q05039F | TGTGTGGGCAGCAATGGA | 59.48 | 111 |
| LITCHI005039 | q05039R | GGGATCTTCGCTTCGGCA | 59.813 | 111 |
| LITCHI006477 | q06477F | GGCTTGTGAGGACTGGGG | 59.647 | 139 |
| LITCHI006477 | q06477R | GCCGCCGGACATATCGAA | 59.972 | 139 |
| LITCHI020852 | q20852F | ACAGTCCTCCGTCTCGCT | 59.966 | 110 |
| LITCHI020852 | q20852R | TGGGTGTCAGTGGGACCA | 60.04 | 110 |
| LITCHI013091 | q13091F | TCGCAATCGCTCTCGTCC | 59.898 | 109 |
| LITCHI013091 | q13091R | TGGGGACGTAGCACCAGA | 59.881 | 109 |
| LITCHI022925 | q22925F | AACATGGTTCCCGGGCTG | 59.964 | 118 |
| LITCHI022925 | q22925R | ACTTGCCGTTGCTGAGGA | 59.171 | 118 |
| LITCHI015108 | q15108F | GGTTGCTTTGCTGGTGGC | 59.97 | 131 |
| LITCHI015108 | q15108R | AGGTGGGTCTCGCTAGGG | 60.043 | 131 |
| LITCHI031057 | q31057F | CACCGTCGTGTCCAAGCT | 59.971 | 86 |
| LITCHI031057 | q31057R | CTTGGAGCCAGTTGCCGA | 59.967 | 86 |
| LITCHI027959 | q27959F | GCCCTCCGTCAAACCTCC | 60.046 | 108 |
| LITCHI027959 | q27959R | ACTCCGATCGCCGTGAAC | 59.819 | 108 |
