# Supplementary figures and images for: Multi-omics elucidation of yellow aril coloration in litchi (Litchi chinensis Sonn.) cultivar ‘Jianjianghongnuo’: coordinated downregulation of flavonoid and carotenoid biosynthetic pathways drives pigment dynamics
Source: Front Plant Sci. 2025 Oct 6;16:1669458. doi: 10.3389/fpls.2025.1669458 (PMC12535983; doi:10.3389/fpls.2025.1669458)

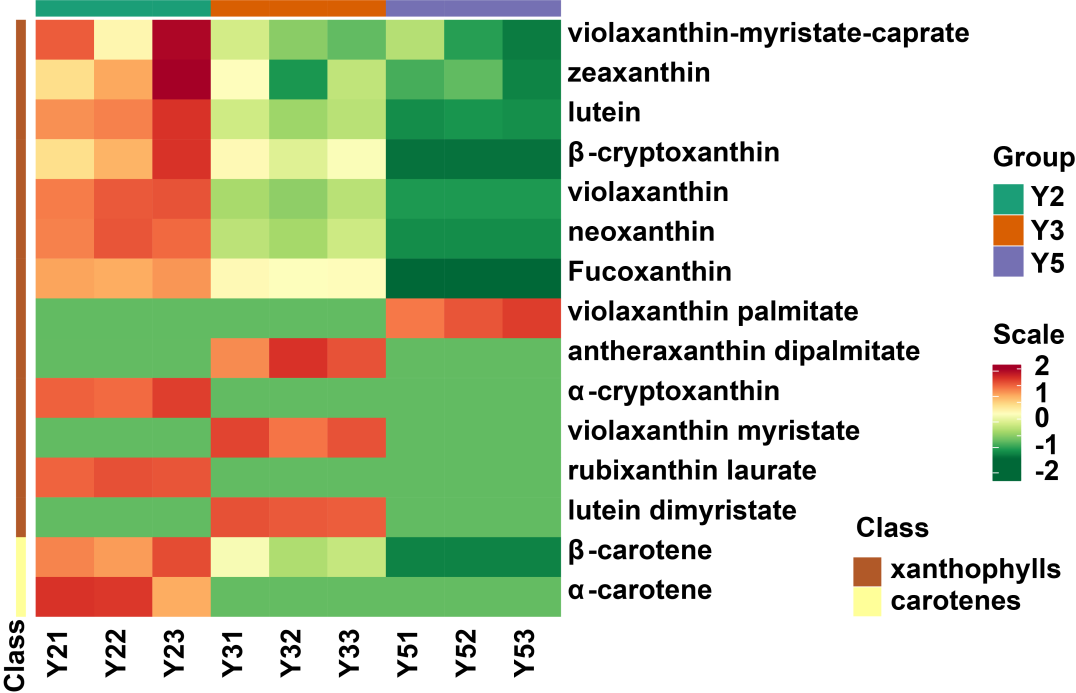


Supplementary Figure S1. Clustered Heatmap of Carotenoid Content

Supplement: Supplementary file 1 [file DataSheet1.zip › 250926Re-submit Supplementary Material/Supplementary Figure S1 Clustered Heatmap of Carotenoid Content.docx]
